# Editorial: Mutational signatures and immune response in cancer

**DOI:** 10.3389/fgene.2023.1215326

**Published:** 2023-05-17

**Authors:** Ilias Georgakopoulos-Soares, Apostolos Zaravinos

**Affiliations:** ^1^ Department of Biochemistry and Molecular Biology, Institute for Personalized Medicine, The Pennsylvania State University College of Medicine, Hershey, PA, United States; ^2^ Department of Life Sciences, School of Sciences, European University Cyprus, Nicosia, Cyprus; ^3^ Cancer Genetics, Genomics and Systems Biology Laboratory, Basic and Translational Cancer Research Center (BTCRC), Nicosia, Cyprus

**Keywords:** mutational signatures, tumor immune microenvironment, biomarkers, immune response, cancer, mutations

Cancer is a disease characterized by the accumulation of somatic mutations. The types of somatic mutations vary, and include substitutions, indels, rearrangements and copy number variations, which are found at different frequencies across different cancer types and patient samples. Mutational signatures represent the mutational processes that are responsible for this accumulation of mutations, and these different types of processes include DNA replication infidelity, carcinogens, including tobacco products and UV light exposure, DNA repair deficiencies, such as mismatch repair and homologous recombination repair deficiencies, and enzymatic DNA editing, among others. The ever-increasing amount of data related to cancer genomes provide a wealth of information for the development of novel insights on mutagenic processes, biological mechanisms involved and opportunities for new therapies. The identification of tumor vulnerabilities also enables patient stratification and the development of targeted therapies, which implore treatment outcomes.

The analysis of the activity of mutational processes in the cancer genome provides insights to the active biological mechanisms that drive it. Furthermore, the tumor mutational burden, which represents the somatic mutations found in a tumor sample, associates with the success of immunotherapies, such as those based on immunomodulators or those that target neoantigens.

The relationship between the tumor microenvironment (TME) and immunological response is another topic of active research. It remains, however, unclear how the tumor’s immune microenvironment influences the accumulation, location and frequency of somatic mutations and how it affects the ability of the immune system to elicit a response against cancer cells.

The collection of works for this Research Topic (RT), includes the characterization of somatic mutations and their association with DNA repair deficiencies and the examination of different biomarkers for cancer prognosis and response to immunotherapies. The RT also provides important insights into the TME and its association with mutations, cancer development and immune response.

In a pertinent study published in this RT, Liu et al. investigated fatty acid metabolism-related genes in soft tissue sarcoma and identified a new score based on fatty acid metabolism, which may be provided as a potential biomarker and treatment strategy in this disease. What’s more, Loizides et al. discuss the role of high immunogenicity, specific immune activation signatures, as well as that of high expression of immunosuppressive genes and high levels of stromal tumor infiltrating lymphocytes (TILs), as key elements of the immune-driven landscape associated with triple negative breast cancer. In another study, Wang et al., the authors developed an angiogenesis-related gene risk assessment model to predict the prognosis of lung adenocarcinoma patients. This model was suggested to help in patient classification and the selection of medications for them. In addition, Zhong et al. (2023) explored the relationship and potential mechanisms between TTN mutation status and immune response, pan-cancer. They constructed and validated a TTN mutation-associated immune prognostic model using four immune-related genes and analyzed the potential mechanisms of this model to predict patient prognosis and response to immunotherapy from an immunological perspective. Additionally, the study by Dai et al. shows that PBRM1 mutation is closely related to immune efficacy and immune microenvironment, including killer cell mediated immunity regulation, cell cytokine production, CD8^+^ T-cell activation and MHC protein binding process. This correlation between PBRM1 mutation and prognosis and immune response, suggests that it may be used as a promising immunotherapeutic signature that could guide clinical management and personalized immunotherapy. Furthermore, Borch et al. explored different tumor associated characteristics for their association with favorable clinical outcome in a diverse cohort of cancer patients treated with immune checkpoint inhibitors. Their study revealed that even across this diverse cohort, patients achieving clinical benefit had significantly higher neoepitope load, higher expression of T cell signatures, and higher PD-L2 expression, which also correlated with improved progression-free and overall survival. Finally, the study by Huang et al. evaluated copy number deletion events as candidate targets across 17 cancer types. The authors identified a broad range of significant genes hit by copy deletion, where RB1, PTEN and CDKN2A were the most significantly deleted genes among all cancer types.

Overall, the studies in this RT contribute to an improved understanding of different mutational patterns in the TME and how they could provide further therapeutic opportunities for cancer patients. We would like to thank all the authors who contributed their original work to our RT and the reviewers for their valuable comments. We also thank the Frontiers Editorial Office for providing us with the opportunity to host this RT.
